# The association of circulating levels of complement-C1q TNF-related protein 5 (CTRP5) with nonalcoholic fatty liver disease and type 2 diabetes: a case–control study

**DOI:** 10.1186/s13098-015-0099-z

**Published:** 2015-11-25

**Authors:** Solaleh Emamgholipour, Nariman Moradi, Maani Beigy, Parisa Shabani, Reza Fadaei, Hossein Poustchi, Mahmood Doosti

**Affiliations:** Department of Clinical Biochemistry, Faculty of Medicine, Tehran University of Medical Sciences, Tehran, Iran; Department of Clinical Biochemistry, Faculty of Medicine, Iran University of Medical Sciences, Tehran, Iran; Students’ Scientific Research Center, Tehran University of Medical Sciences, Tehran, Iran; Liver and Pancreatobiliary Diseases Research Center, Digestive Diseases Research Institute, Tehran University of Medical Sciences, Tehran, Iran

**Keywords:** Type 2 diabetes mellitus, Nonalcoholic fatty liver disease, CTRP5, Insulin resistance

## Abstract

**Background:**

It is well-established that nonalcoholic fatty liver disease (NAFLD) is associated with type 2 diabetes mellitus (T2DM). Complement-C1q TNF-related protein 5 (CTRP5) is a novel adipokine involved in the regulation of lipid and glucose metabolism. We aimed to assess plasma levels of CTRP5 in patients with NAFLD (n = 22), T2DM (n = 22) and NAFLD with T2DM (NAFLD + T2DM) (n = 22) in comparison with healthy subjects (n = 21) and also to study the association between CTRP5 levels and NAFLD and diabetes-related parameters.

**Methods:**

All subjects underwent anthropometric assessment, biochemical evaluation and liver stiffness (LS) measurement. Insulin resistance (IR) was determined by the homeostasis model assessment (HOMA). Plasma CTRP5 levels were measured by enzyme-linked immunosorbent assay.

**Results:**

We found significantly lower plasma levels of CTRP5 in patients with NAFLD + T2DM, NAFLD and T2DM (122.52 ± 1.92, 124.7 ± 1.82 and 118.31 ± 1.99 ng/ml, respectively) in comparison with controls (164.96 ± 2.95 ng/ml). In the whole study population, there was a significant negative correlations between CTRP5 and body mass index (r = −0.337; p = 0.002), fasting blood glucose (FBG) (r = −0.488; p < 0.001), triglyceride (TG) (r = −0.245; p = 0.031), HOMA-IR (r = −0.492; p < 0.001), insulin(r = −0.338; p = 0.002), LS (r = −0.544; p < 0.001), alanine aminotransferase (ALT) (r = −0.251; p = 0.027), waist-to-hip ratio (WHR) (r = −0.352; p = 0.002) and waist circumference (WC) (r = −0.357; p = 0.001). After adjustment for BMI, decrease in circulating levels of CTRP5 remained as a significant risk factor for NAFLD, T2DM and NAFLD + T2DM. The receiver operating characteristic (ROC) curves of circulating CTRP5 in predicting NAFLD and T2DM demonstrated an area under the curve (AUC) of 0.763 in T2DM, and 0.659 in NAFLD + T2DM.

**Conclusions:**

It appears that the decreased levels of CTRP5 contribute to the increased risk of T2DM and NAFLD.

**Electronic supplementary material:**

The online version of this article (doi:10.1186/s13098-015-0099-z) contains supplementary material, which is available to authorized users.

## Background

Nonalcoholic fatty liver disease (NAFLD) encompasses a range of clinicopathological conditions varying, from simple steatosis alone to nonalcoholic steatohepatitis, which could ultimately lead to the development of cirrhosis and hepatocellular carcinoma [1]. NAFLD is recognized as the major hepatic component of metabolic syndrome, which affects a considerable proportion of patients with obesity and type 2 diabetes mellitus (T2DM) [2–4]. Accumulating evidence indicates a strong association of insulin resistance and obesity with NAFLD pathogenesis [5, 6], although the precise mechanism remains unclear.

One possible mechanism is the increase in production of proinflammatory cytokines (e.g. interleukine-6 and tumor necrosis factor alpha and change in secretion of several adipokines (e.g. visfatin, resistin, leptin and adiponectin), which makes conditions favorable for steatosis development [7–10]. It has been reported that circulating adiponectin decreases in patients with NAFLD. Also, plasma adiponectin level is inversely correlated with hepatic fat stores and insulin resistance [11].

The members of the C1q/TNF-related protein (CTRP) family have recently been reported as proteins that share functional and structural similarity to adiponectin [12–14].

Up to now, 15 CTRP family members have been identified that play important roles in energy homeostasis and inflammation [12–14]. The involvement of CTRP family members in the pathogenesis of several metabolic diseases such as T2DM, obesity and hepatic staetosis are recently beginning to be appreciated. For example, overexpression of CTRP1 caused improvement of insulin sensitivity in transgenic mice [15]. There is also evidence that transgenic animal models overexpressing CTRP3 are resistance to diet-induced steatosis and have low hepatic triglyceride content [16]. In addition, overexpression of CTRP9 in mice reduced hepatic and skeletal muscle triglyceride levels and improved hepatic steatosis in diet-induced obesity [17]. More recent clinical studies have reported that diabetic patients [18] and subjects with metabolic syndrome [19] have higher serum levels of CTRP1 than normal controls. Another study demonstrated that CTRP3 levels increase in metabolic syndrome patients and are strongly related to cardiometabolic parameters [20]. Our recent data showed that circulating levels of CTRP1 are significantly increased in patients with NAFLD and T2DM compared to healthy subjects [21].

Among the CTRP family members, CTRP5 has been demonstrated to be an important molecule related to metabolism regulation [22]. CTRP5 is a protein with 243-amino acids, consisting of N-terminal signal peptide followed by a collagen repeat, and a C-terminal globular domain [23, 24]. This protein is expressed by many tissues including spleen, uterus, testis, brain, retinal pigment, myocytes, and adipocytes, particularly in the stromal vascular cell fraction [25, 26] and was initially recognized as a molecule involved in late-onset macular degeneration and long anterior lens zonules [25]. It has been shown that CTRP5 induces phosphorylation of AMP-activated protein kinase (AMPK), thereby stimulating glucose uptake and fatty acid oxidation [22]. Moreover, circulating CTRP5 is elevated in animal models of obesity-associated diabetes such as Otsuka Long-Evans Tokushima Fatty (OLETF) rats, ob/ob mice, and db/db mice [22]. It has been suggested that CTRP5 might be a human adipokine which circulates in large quantities in serum [27]. The single nucleotide polymorphism in 3′-untranslated region of CTRP5 is also strongly associated with metabolic syndrome in Japanese people [28].

The role of CTRP5 in regulation of lipid and glucose metabolism and its relationship with parameters related to energy metabolism have been shown in several experimental studies [22, 29] and in a limited number of human studies [27, 30, 31]. However, to our knowledge, no study has addressed the association of CTRP5 levels with NAFLD and metabolic- related profile in humans. It is also evident that T2DM is significantly associated with NAFLD pathogenesis. Hence, we aimed to investigate the circulating levels of CTRP5 in patients with NAFLD, T2DM and NAFLD with T2DM (NAFLD + T2DM) in comparison with healthy subjects. We also intended to evaluate the possible association of CTRP5 level with several NAFLD and diabetes-related parameters.

## Subjects and methods

### Study population

A total of 87 subjects (all men) aged between 43 and 72 years, were recruited for this case–control study as described previously [21]. The participants were selected among individuals who attended the outpatient clinic of Shariati Hospital, Tehran, Iran from March 2012 until November 2013. The control group was selected from accompanying people of patients. The study subjects were categorized into healthy subjects (controls) (n = 21), NAFLD patients (n = 22), NAFLD + T2DM patients (n = 22) and T2DM patients (n = 22).

T2DM diagnosis was based on American Diabetes Association (ADA) criteria [32]. It should be mentioned that 6 patients with T2DM and 3 patients with NAFLD + T2DM were receiving anti-diabetic drugs. The mean duration of diabetes was 1.66 ± 0.65 years.

The participants with evidence of viral or autoimmune hepatitis, Wilson’s disease, primary biliary cirrhosis, haemochromatosis, congenital cardiac disease, infectious disease, acute or chronic renal failure, malignancies, type 1 diabetes mellitus and also a history of alcohol consumption of >30 g/day, were excluded from the study. In addition, all patients were free from taking medication causing steatosis (i.e., corticosteroids, valproic acid, amiodarone, estrogens, tamoxifen, amiodarone, valproic acid, diltiazem). Also, 2 patients with NAFLD, 6 ones with T2DM, and 6 patients with NAFLD + T2DM were receiving antihypertensive drugs. Diagnosis of NAFLD was established by a physician using a routine abdominal ultrasonography. Liver stiffness (LS), a noninvasive assessment of liver fibrosis, was measured by a Transient Elastogeraphy (Fibroscan^®^ France). The study was approved by Ethics Committee of the Tehran University of Medical Sciences (TUMS). Informed written consent was signed by all subjects before their participation in the study.

### LS assessment

LS was measured by transient elastography using the FibroScan^®^ 502 machine (EchoSense, Paris, France, 5 MHz) as described previously [21]. Briefly, based on the manufacturer’s guidelines the M probe was used when the thoracic perimeter less than 110 cm, and the XL probe was used when the thoracic perimeter was 110 cm and above. The measurements were repeated at least 10 times for each individual and the median value was calculated. If the inter-quartile range (IQR) was less than 30 % of the median reading, values were considered representative of LS.

### Anthropometric and clinical characterization

All participants underwent anthropometric assessment, ultrasonographic and biochemical evaluation. A self-reported and standard questionnaire was used to collect demographic and socioeconomic characteristics, medical history and drug use from each participant.

The body mass index (BMI) was computed by dividing the weight (in kilograms) with the square of height (in meters). Waist circumference (WC) was measured to the nearest 0.1 cm at the level of the iliac crest with a flexible inch tape while the subject was at minimal respiration. Hip measurements were taken at the maximum circumference of the buttocks. Waist-to-hip ratio (WHR) was calculated as waist circumference (in centimeters) divided by hip circumference (in centimeters). Homeostasis model assessment of insulin resistance (HOMA-IR) was calculated as follows: [fasting blood glucose (mg/dL)] × [fasting blood insulin (µU/mL)/405]. Systolic and diastolic blood pressures of all participants were measured after 15 min rest in a sitting position with a manual sphygmomanometer.

### Biochemical and laboratory measurements

Venous blood was collected following an overnight fasting and divided into two aliquots, in clot activator tube and vacutainer containing EDTA, in order to biochemical analyses and CTRP5 measurement, respectively. Samples were centrifuged and serum and plasma were separated and either used immediately or stored at −80 °C until assayed. Fasting blood glucose (FBG) was measured using the glucose oxidase method. Insulin was assessed using enzyme linked immunosorbent assay (ELISA) kit (Monobind Inc., USA). Serum triglycerides (TG), high-density lipoprotein cholesterol (HDL-C), low-density lipoprotein cholesterol (LDL-C) and total cholesterol (TC), creatinin and urea were assessed using a commercially available kit (Pars Azmoon, Tehran, Iran). The levels of alanine amino transferase (ALT), aspartate amino transferase (AST), gamma glutamyl transferase (γ-GT) and alkaline phosphatase (ALP) were measured using enzymatic colorimetric assays (Pars Azmoon kit, Tehran, Iran). The blood cell-related indicators red blood cell (RBC), white blood cells (WBC), Hemoglobin, platelets, mean corpuscular volume (MCV), and red cell distribution width (RDW) were evaluated by automatic analyzer. The AST to platelet ratio index (APRI), as a noninvasive marker to assess liver fibrosis, was calculated as AST (IU/l)/(upper limit of normal)/platelet count (×10^9^/L) ×100 [33]. It should be noted that some data from this study were published recently [21].

### Plasma CTRP5 measurement

Plasma levels of CTRP5 were determined by immunoassay using Cayman system kit according to manufacturer’s protocol. The inter-assay variability and intra-assay variability were 6.975 and 6.3 %, respectively.

### Statistical analysis

Data analysis was performed using SPSS 16 (SPSS, Chicago, IL, USA). Descriptive analysis was applied and normality was tested using the Shapiro–Wilk test for all quantitative variables. The data of variables with normal distribution are expressed as mean ± standard error of the means (SEM), and data of variables without normal distribution are expressed as median ± interquartile ranges (IQR). For data with normal distribution, comparisons among the four groups were done by the one-way ANOVA.

When significant differences were found, the Bonferroni post hoc test was used for multiple comparisons.

For non-normally distributed variables, comparisons among the four groups were determined with the Kruskal–Wallis test. When significant differences were found, differences between two independent groups were determined by the Mann–Whitney U test. We used the Bonferroni correction to reduce the probability of spurious positives in multiple testing.

We also conducted multinomial logistic regression to investigate the risk of diseases (NAFLD, T2DM, and NAFLD + T2DM) regarding CTRP5, BMI, WC, hip, and WHR. Receiver operating characteristic (ROC) curve was also plotted using SPSS 16 to reflect the sensitivity and specificity of CTRP5 in order to evaluate their ability to differentiate the investigated diseases. The comparison of the area under the curve (AUC) was performed by a p value <0.05. The greater AUC represents the higher diagnostic value for CTRP5 to differentiate the diseases.

## Results

The anthropometric parameters and biochemical characteristics of patients and control subjects are presented in Table 1.

**Table 1:** Anthropometric and laboratory characteristics of healthy subjects, NAFLD, T2DM and NAFLD + T2DM

Characteristics	Healthy subjects (N = 21)	NAFLD (N = 22)	T2DM (N = 22)	NAFLD + T2DM (N = 22)	Total difference *p* value
Age, years	51 (48–60)	51 (48–55)	57.5 (47–60)	52 (45–57)	ns
WC, cm	93.29 ± 2.14	104.95 ± 1.49	100.43 ± 2.38	109.61 ± 2.23	<0.001
Hip, cm	99.29 ± 1.23	105.32 ± 1.2	100.77 ± 1.51	107.73 ± 73	<0.001
WHR, –	0.94 ± 0.01	1.00 ± 0.01	0.99 ± 0.02	1.02 ± 0.02	<0.001
BMI, kg/m^2^	24.76 ± 0.80	29.18 ± 0.50	27.28 ± 0.91	30.61 ± 0.84	<0.001
FBG, mg/dL	89.46 (84.10–96.56)	95.82 (90.10–100.70)	129.95 (123.20–175.20)	155 (127.00–187.95)	<0.001
Insulin, µU/mL	3.5 (2.7–4.9)	9.6 (9.2–12)	6.35 (2.2–8.9)	8.5 (6.3–10.6)	<0.001
HOMA-IR, –	0.75 (0.56–1.23)	2.32 (1.89–2.89)	2.46 (0.91–3.17)	2.64 (1.99–5.51)	<0.001
TG, mg/dL	114.65 (89.95–154)	143.95 (109.1–164.8)	138.7 (105.3–163.7)	165.6 (113.75–241.05)	ns
TC, mg/dL	190.01 ± 6.69	201.45 ± 7.64	195.84 ± 9.67	191.42 ± 16.17	ns
HDL-C, mg/dL	54.39 ± 2.78	48.61 ± 2.16	54.22 ± 2.88	49.79 ± 3.98	ns
LDL-C, mg/dL	111.65 ± 6.41	117.73 ± 7.75	113.76 ± 7.85	109.22 ± 10.53	ns
LDL-C/HDL-C, –	2.12 ± 0.14	2.42 ± 0.15	2.13 ± 0.13	2.04 ± 0.19	ns
TC/HDL-C, –	3.65 ± 0.16	4.24 ± 0.17	3.69 ± 0.14	3.59 ± 0.30	ns
Urea nitrogen, mg/dL	28.13 ± 1.17	32.29 ± 1.90	30.88 ± 1.33	30.98 ± 2.42	ns
Creatinin, mg/dL	1.27 ± 0.04	1.29 ± 0.04	1.25 ± 0.04	1.10 ± 0.08	ns
AST, U/L	17.1 (15.2–18.3)	22.1 (18.3–30.9)	16.55 (14.6–19.00)	24.6 (21.55–28.1)	<0.001
ALT, U/L	15.3 (12.55–18.4)	28.9 (22.5–44.5)	15.6 (12.7–21.6)	41.75 (32.9–52.9)	<0.001
γ-GT, U/L	19.9 (16.26–23.79)	28.96 (24.3–36.1)	23.49 (19.79–36.74)	36.97 (27.79–72.65)	<0.001
ALP, U/L	224.5 (202–249)	231.5 (195–278)	243 (190–316)	228.5 (186.5–271)	ns
SBP, mmHg	127.65 ± 4.43	130.84 ± 4.49	136.79 ± 4.53	137.02 ± 4.40	ns
DBP, mmHg	78.15 ± 2.37	84.39 ± 3.76	79.95 ± 2.50	80.70 ± 2.10	ns
LS, kPa	2.33 ± 0.48	5.46 ± 0.37	4.77 ± 0.32	7.00 ± 0.51	<0.001
RBC, ×10^12^/L	4.66 ± 0.11	4.9 ± 0.09	4.96 ± 0.09	4.96 ± 0.13	ns
Platelet, ×10^9^/L	221.05 ± 11.63	223.91 ± 10.65	224.64 ± 9.21	240.1 ± 8.27	ns
APRI, –	0.22 (0.15–0.25)	0.28 (0.19–0.41)	0.18 (0.15–0.21)	0.24 (0.22–0.30)	<0.05
WBC, ×10^9^/L	5.5 (5.2–6.5)	5.5 (5.1–6.9)	6.6 (5.7–7.6)	6.65 (5.55–7.55)	ns
Hemoglobin, g/dl	14 (12–15)	14 (14–15)	14.5 (12–16)	14 (12–15.5)	ns
RDW, %	14 (14–15)	14 (14–14)	14.5 (14–15)	14 (14–15)	ns
MCV, fL	87 (86–89)	85 (83–88)	84.5 (82–88)	82 (79–89)	ns

Continuous variables with normal and non-normal distribution were described as mean ± SEM and median (IQR), respectively

*NAFLD* nonalcoholic fatty liver disease, *T2DM* type 2 diabetes mellitus, *n* number, *WC* waist circumference, *WHR* waist-to-hip ratio, *BMI* body mass index, *FBG* fasting blood glucose, *HOMA-IR* homeostasis model assessment of insulin resistance, *TG* triglycerides, *TC* total cholesterol, *HDL-C* high-density lipoprotein cholesterol, *LDL-C* low-density lipoprotein cholesterol, *ALT* alanine amino transferase, *AST* aspartate amino transferase, *ɤ-GT* gamma glutamyl transferase, *ALP* alkaline phosphatase, *LS* liver stiffness, *SBP* systolic blood pressure, *DBP* diastolic blood pressure, *RBC* red blood cell, *WBC* white blood cell, *MCV* mean corpuscular volume, *RDW* red cell distribution width, *APRI* aspartate amino transferase to platelet ratio index, *ns* non-significant

No significant difference was found among individuals with NAFLD, T2DM, and NAFLD + T2DM groups in terms of age, TG, TC, HDL-C, LDL-C, LDL-C/HDL-C, cholesterol/HDL-C, ALP, urea, creatinin, systolic blood pressure (SBP), diastolic blood pressure (DBP), RBC, WBC, MCV, RDW, platelets and hemoglobin.

Based on one-way ANOVA; WC, hip, WHR, BMI, and LS were significantly different among the all studied groups. In addition, the Kruskal–Wallis test showed that there were significant differences in HOMA-IR, FBG, insulin, APRI, AST, ALT, and γ-GT among the all studied groups. Additional file 1: Table S1 shows the results of post hoc analysis for WC, hip, WHR, BMI, LS, HOMA-IR, FBG, insulin, APRI, AST, ALT, and γ-GT which were significantly different among the groups.

**Fig. 1:**
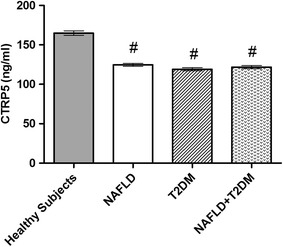
The CTRP5 levels in patients with NAFLD, T2DM, and NAFLD + T2DM in comparison with control group (^#^ *p* values < 0.001). Each *bar* represents mean ± SEM

The plasma concentration of CTRP5 was depicted in Fig. 1. This adipokine was found to be significantly (p < 0.001) lower in patients with NAFLD + T2DM compared with the controls (122.52 ± 1.92 ng/ml in NAFLD + T2DM patients, 164.96 ± 2.95 ng/ml in control subjects). In addition, the plasma concentration of CTRP5 was significantly lower in NAFLD patients and T2DM subjects compared with the controls (124.7 ± 1.82 ng/ml in NAFLD; p < 0.001 vs. controls; 118.31 ± 1.99 ng/ml in T2DM; p < 0.001 vs. controls).

Results of multinomial logistic regression analysis demonstrated the main effect of plasma level of CTRP5, WC, hip and WHR and BMI on the risk of all three conditions (Table 2). From the diagnostic standpoint, along with every 1 ng/ml decrease in CTRP5 level, NAFLD risk was significantly (p < 0.001) increased 662.25 times. Moreover, the risk of T2DM and NAFLD + T2DM were significantly (p < 0.001) increased 714.28 and 684.93 times, respectively, along with every 1 ng/ml decrease in CTRP5 level (Table 2a). After adjustment for WC (Table 2c), hip (Table 2f), WHR (Table 2i) and BMI (Table 2l), decrease in circulating levels of CTRP5 remained as a significant risk factor for NAFLD, T2DM and NAFLD + T2DM although BMI-adjusted odd ratio (OR) and hip-adjusted OR were decreased by almost tenfold in comparison with before adjustment.

**Table 2:** Multinomial logistic regression for the association of CTRP5 (a) WC (b) hip (e), WHR (h) and BMI (k) with outcome risk of NAFLD, T2DM, and NAFLD + T2DM

Groups	B	SE	Wald	*p* value	Odds ratio	95 % confidence interval for OR	Correct prediction (%)	
a. Risk of outcomes along with each unit increase in CTRP5								
NAFLD	−6.495	0.036	32,216.808	<0.001	0.00151	0.00141–0.00162	40.9	
T2DM	−6.571	0.039	28,394.414	<0.001	0.00140	0.00130–0.00151	66.7	
NAFLD + T2DM	−6.531	0	–	–	0.00146	0.00146–0.001461	26.1	
b. Risk of outcomes along with each unit increase in WC								
NAFLD	0.126	0.038	10.756	0.001	1.134	1.052–1.223	22.7	
T2DM	0.071	0.033	4.612	0.032	1.074	1.006–1.146	27.3	
NAFLD + T2DM	0.191	0.044	18.743	<0.001	1.210	1.110–1.319	54.5	
c. Adjustment of CTRP5 risk for WC^†^								
NAFLD	−6.486	0.039	28,258.994	<0.001	0.00152	0.00141–0.00164	47.6	
T2DM	−6.555	0.043	23,663.923	<0.001	0.00142	0.00130–0.00154	52.4	
NAFLD + T2DM	−6.522	<0.001	–	–	0.001	0.001–0.001	47.4	
d. Interaction risk (CTRP5 × WC) for each unit increase in CTRP5 and each unit increase of WC^†^								
NAFLD	0.0	0.0	11.289	0.001	0.999	0.99–1	52.4	
T2DM	−0.001	0.0	20.162	<0.001	0.999	0.98–0.99	57.1	
NAFLD + T2DM	0.0	0.0	8.270	0.004	0.999	0.99–1	10.5	
e. Risk of outcomes along with each unit increase in hip								
NAFLD	0.169	0.058	8.403	0.004	1.184	1.056–1.327		18.2
T2DM	0.037	0.049	0.587	0.444	1.038	0.943–1.143		22.7
NAFLD + T2DM	0.246	0.065	14.306	<0.001	1.279	1.126–1.453		54.5
f. Adjustment of CTRP5 risk for hip^†^								
NAFLD	−4.922	0.038	16,351.610	<0.001	0.00728	0.00675–0.00785		47.6
T2DM	−4.994	0.043	13,373.185	<0.001	0.00677	0.00622–0.00737		52.4
NAFLD + T2DM	−4.953	0	–	<0.001	0.00706	0.00706–0.00706		42.1
g. Interaction risk (CTRP5 × hip) for each unit increase in CTRP5 and each unit increase of hip †								
NAFLD	−0.002	0.0005	13.028	<0.001	0.9983	0.9974–0.9992		66.7
T2DM	−0.003	0.001	23.169	<0.001	0.9974	0.9964–0.9984		61.9
NAFLD + T2DM	−0.0002	0.0005	13.013	<0.001	0.9983	0.9973–0.9992		0
h. Risk of outcomes along with each unit increase in WHR								
NAFLD	17.519	6.128	8.171	0.004	40,570,699.7	246.43–6.67 × 10^12^		63.6
T2DM	16.808	6.191	7.372	0.007	19,938,468.5	107.2–3.7 × 10^12^		0
NAFLD + T2DM	24.517	6.609	13.760	<0.001	44,428,129,710.6	105147.7–1.8 × 10^16^		57.1
i. Adjustment of CTRP5 risk for WHR^†^								
NAFLD	−6.519	0.039	28,193.410	0.001	0.00147	0.00136–0.00159		61.9
T2DM	−6.588	0.041	25,291.299	0.001	0.00137	0.00126–0.00149		42.1
NAFLD + T2DM	−6.559	<0.001	–	–	0.00141	0.00141–0.00141		38.9
j. Interaction risk (CTRP5 × WHR) for each unit increase in CTRP5 and each unit increase of WHR^†^								
NAFLD	−0.161	0.043	14.026	<0.001	0.851	0.783–0.926		61.9
T2DM	−0.193	0.046	17.424	<0.001	0.824	0.753–0.903		52.6
NAFLD + T2DM	−0.151	0.043	12.6	<0.001	0.860	0.791–0.935		0
k. Risk of outcomes along with each unit increase in BMI								
NAFLD	0.338	0.101	11.155	0.001	1.403	1.150–1.711		31.8
T2DM	0.173	0.087	3.976	0.046	1.189	1.003–1.409		27.3
NAFLD + T2DM	0.500	0.117	18.150	<0.001	1.648	1.310–2.074		63.6
l. Adjustment of CTRP5 risk for BMI levels^†^								
NAFLD	−4.243	0.038	12,261.550	<0.001	0.0144	0.0133–0.0155		47.6
T2DM	−4.308	0.043	10,259.458	<0.001	0.0134	0.0124–0.0146		57.1
NAFLD + T2DM	−4.283	<0.001	–	–	0.0138	0.0138–0.0138		57.9
m. Interaction risk (CTRP5 × BMI) for each unit increase in CTRP5 and each unit increase of BMI								
NAFLD	−0.001	0.001	5.166	0.023	0.999	0.997–1.000		57.1
T2DM	−0.003	0.001	14.793	<0.001	0.997	0.996–0.999		61.9
NAFLD + T2DM	0.000	0.001	2.864	0.091	0.999	0.998–1.000		0

Adjustment for risk of CTRP5 for the aforesaid outcome diseases by controlling for WC (c) hip (f), WHR (i) and BMI (l) The interactive risk of increasing CTRP5 levels with WC (d) hip (g), WHR (j) and BMI (m) for the aforesaid outcome diseases

*NAFLD* non-alcoholic fatty liver disease, *T2DM* type 2 diabetes mellitus, *CTRP5* complement-C1q TNF-related protein 5, *BMI* body mass index, *WC* waist circumference, *WHR* waist-to-hip ratio

^†^Likelihood Ratio Test: p value < 0.0001

Based on logistic regression and significant likelihood ratio test (p < 0.0001); decreasing plasma levels of CTRP5 showed a slight significant interaction with increasing WC values (Table 2d), increasing hip values (Table 2g), increasing WHR values (Table 2j) and increasing BMI values (Table 2m) for disease risk.

**Fig. 2:**
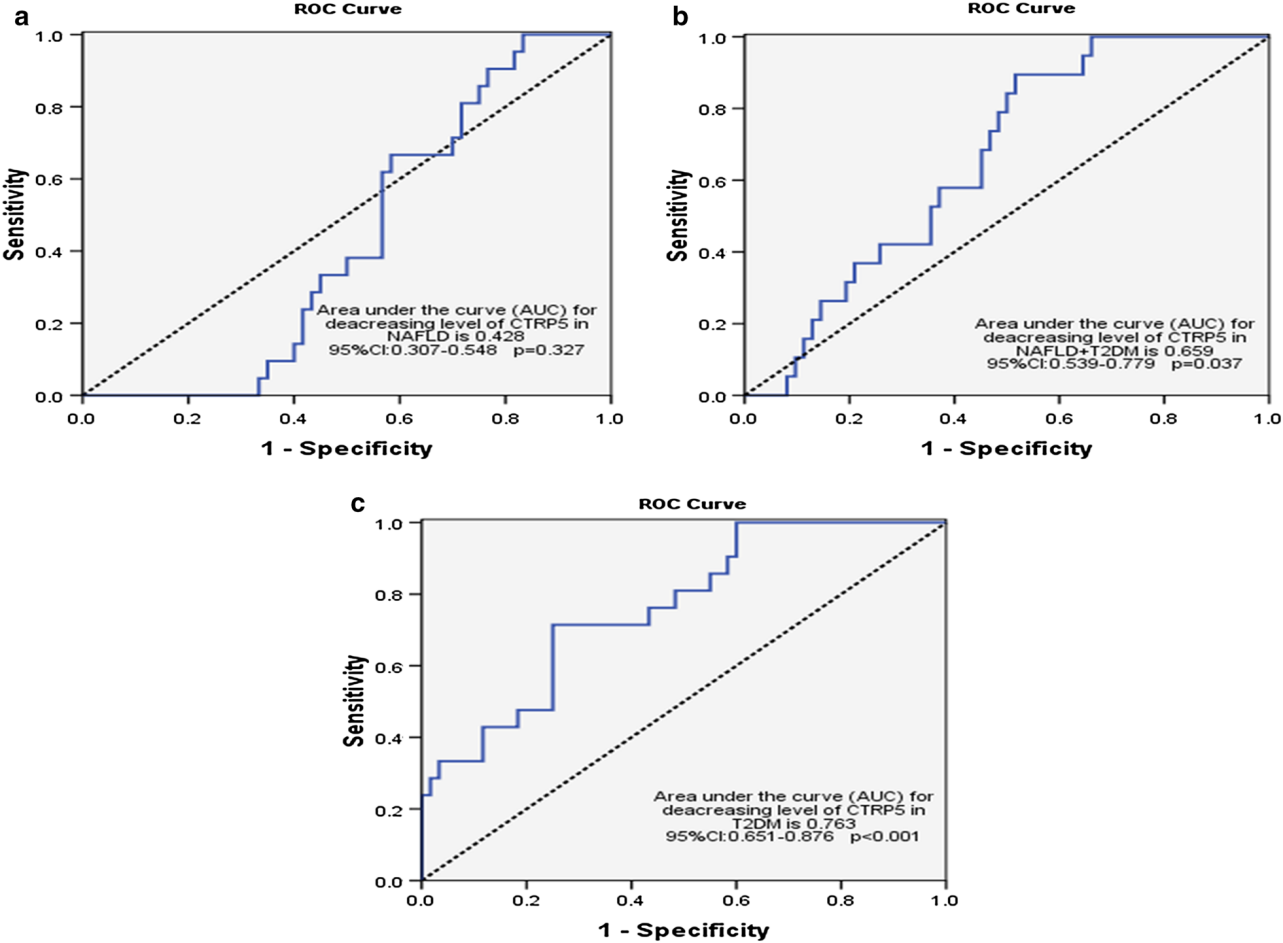
Receiver operating characteristic (ROC) curves for diagnosis NAFLD (**a**), NAFLD + T2DM (**b**), and T2DM patients (**c**) by CTRP5 levels. The comparison of the area under the curve (AUC) was performed by a p value <0.05

**Fig. 3:**
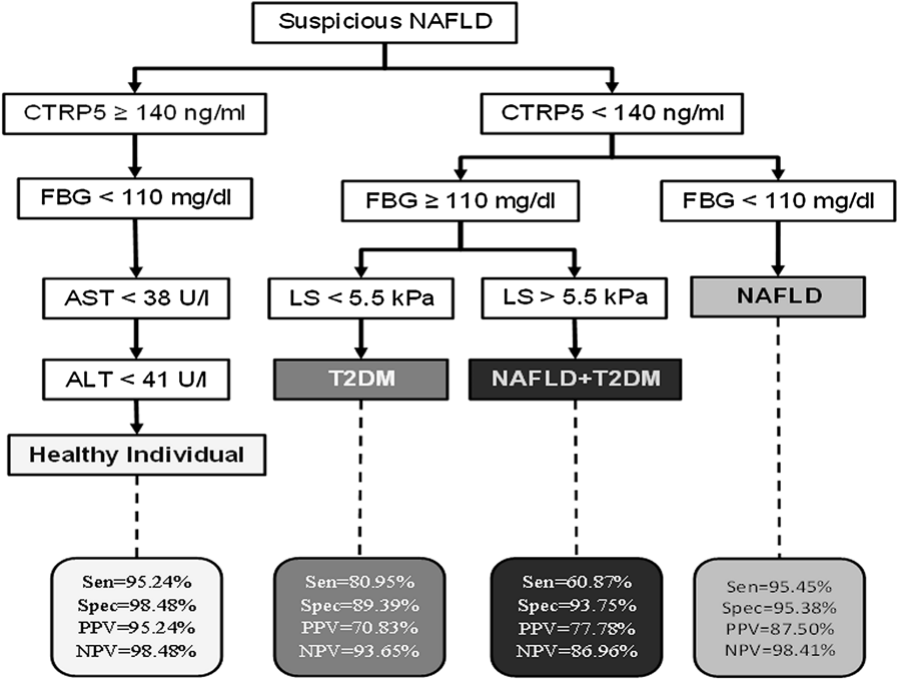
Diagnostic algorithm for differentiating NAFLD, T2DM, NAFLD + T2DM and healthy individuals. *CTRP5* complement-C1q TNF-related protein 5, *FBG* fasting blood glucose, *AST* aspartate aminotransferase, *ALT* alanine aminotransferase, *LS* liver stiffness, *Sen* sensitivity, *Spec* specificity, *NPV* negative predictive value, *PPV* positive predictive value

The ROC curves of CTRP5 circulating levels in predicting NAFLD, T2DM, and NAFLD + T2DM demonstrated an area under the curve (AUC) of 0.428 in NAFLD, 0.659 in NAFLD + T2DM and 0.763 in T2DM. Interestingly, the ROC curve for decreasing levels of CTRP5 for T2DM (Fig. 2c) exhibited a good diagnostic feature. However, the ROC curve for decreasing levels of CTRP5 for NAFLD and NAFLD + T2DM (Fig. 2a, b) did not represent a good diagnostic feature, mainly because of poor specificity. To overcome this disadvantage, we provided a diagnostic algorithm (Fig. 3) based on CTRP5 levels and other laboratory measurements. Based on this diagnostic algorithm, CTRP5 <140 ng/ml and FBG <110 mg/dl showed astonishing diagnostic capabilities for NAFLD (sensitivity = 95.45 %; specificity = 95.38 %; positive predictive value (PPV) = 87.5 %, negative predictive value (NPV) = 98.41 %). We also found that CTRP5 <140 ng/ml, FBG ≥110 mg/dl, and LS <5.5 kPa might be a plausible criteria to differentiate T2DM individuals from NAFLD, NAFLD + T2DM and healthy individuals (sensitivity = 80.95 %; specificity = 89.35 %; PPV = 70.83 %, NPV = 93.65 %). The diagnostic algorithm (Fig. 3) showed CTRP5 < 140 ng/ml, FBG ≥ 110 mg/dl, and LS >5.5 kPa is a specific criteria to differentiate NAFLD + T2DM individuals from NAFLD, T2DM and healthy individuals (sensitivity = 60.87 %; specificity = 93.75 %; PPV = 77.78 %, NPV = 86.96 %).

**Fig. 4:**
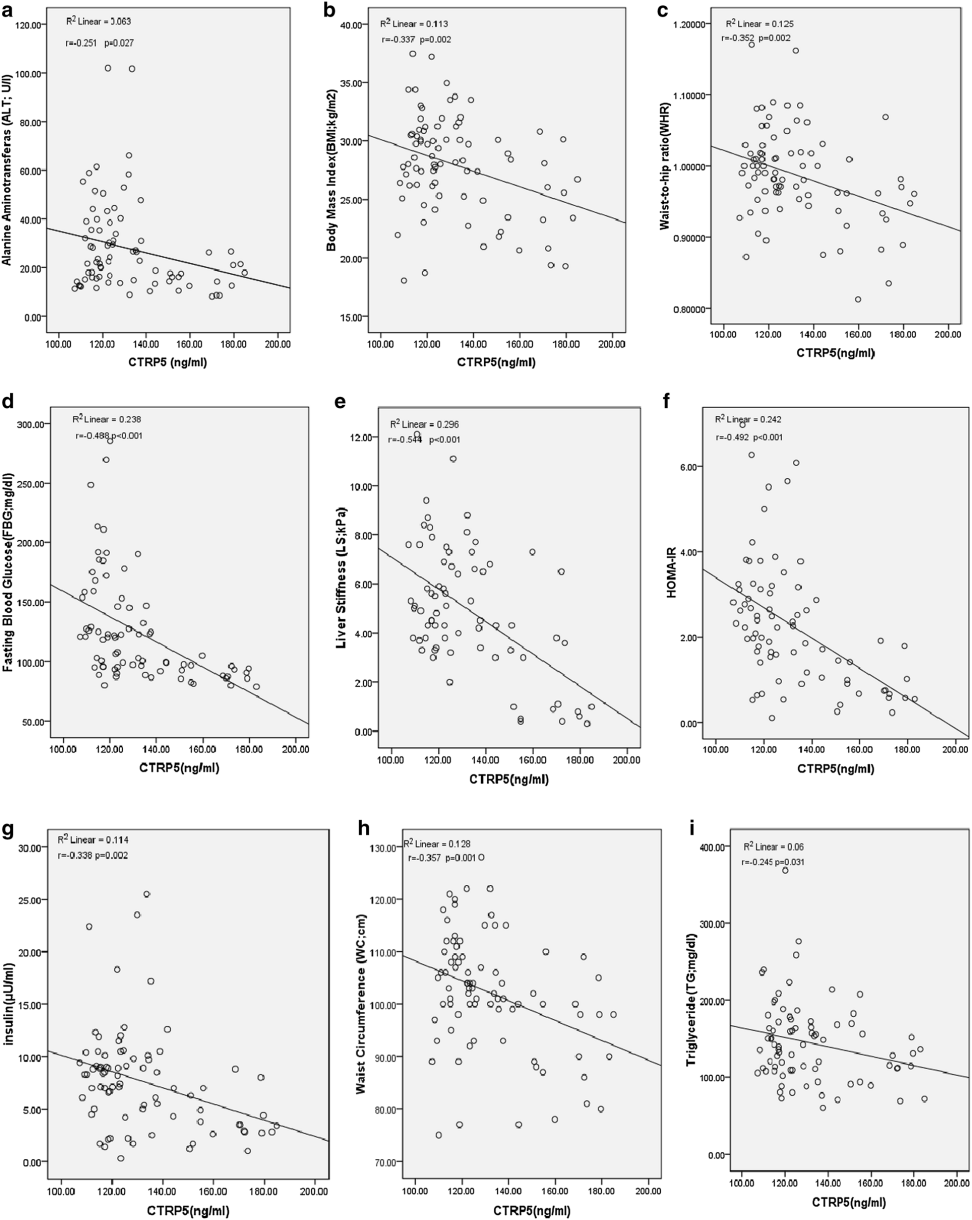
Graphic representation of correlations between CTRP5 levels and serum ALT (**a**), BMI (**b**), WHR (**c**), FBG (**d**), LS (**e**), HOMA-IR (**f**), Insulin (**g**), WC (**h**) and TG (**i**). Correlation coefficient (r) and p values are calculated by the Pearson correlation method

In the whole study population, CTRP5 circulating levels demonstrated a significant negative correlation with BMI (r = −0.337; p = 0.002), WHR (r = −0.352; p = 0.002) and WC (r = −0.357; p = 0.001) (Fig. 4b, c, h).

We also found a significant inverse correlation between CTRP5 levels and FBG (r = −0.488; p < 0.001), HOMA-IR (r = −0.492; p < 0.001), insulin (r = −0.338; p = 0.002) and TG (r = −0.245; p = 0.031) (Fig. 4d, f, g, i).

Moreover, a significant inverse correlation was observed between plasma level of CTRP5 and ALT (r = −0.251; p = 0.027) and also between CTRP5 and LS (r = −0.544; p < 0.001) (Fig. 4a, e).

We also analyzed the possible effects of anti-diabetic and anti-hypertensive medications on plasma levels of CTRP5. We observed no significant differences regarding the interaction of status (groups) × anti-diabetic treatments or status × anti-hypertensive medications (data not shown), hence we conducted analysis of covariance (ANCOVA) to remove possible effects of anti-diabetic and anti-hypertensive medications on plasma levels of CTRP5 (Table 3). As shown in this table, CTRP5 levels were significantly different between the four studied groups after removing the possible effects of aforesaid medications.

**Table 3:** A full factorial model of ANCOVA to adjust the possible effect of anti-diabetic and anti-hypertensive drugs on circulating levels of CTRP5 in controls, NAFLD, T2DM and NAFLD + T2DM patients

Status	Status	Mean difference (I − J) ± SEM	p value	95 % confidence interval for difference	
				Lower bound	Upper bound
Control	NAFLD	40.208 ± 3.164	0.000	31.657	48.759
	T2DM	45.197 ± 3.338	0.000	36.176	54.219
	NAFLD + T2DM	42.926 ± 3.293	0.000	34.028	51.824

*NAFLD* non-alcoholic fatty liver disease, *T2DM* type 2 diabetes mellitus, *CTRP5* complement-C1q TNF-related protein 5, *SEM* standard error of mean

## Discussion

More recently, the importance of CTRP family members in the development of metabolic disorders is beginning to emerge from several studies [20, 30, 34]. However, the clinical relevance of CTRP5 in NAFLD and other diabetes-related disorders is yet unknown. To our knowledge, this is the first study reporting a strong association of circulating levels of CTRP5 with NAFLD and T2DM in humans.

The main findings of this study are as follows: (1) circulating levels of CTRP5 in patients with NAFLD, T2DM and NAFLD + T2DM were markedly lower compared to the controls; (2) inverse correlations were observed between circulating CTRP5 levels and some parameters of glucose metabolism (FBG, insulin and HOMA-IR), fat mass (BMI, WC and WHR) and lipid metabolism (TG) in the whole population; (3) an inverse correlation was found between circulating level of CTRP5 and LS and also between CTRP5 and ALT; (4) decreased circulating CTRP5 levels were strongly associated with the increased risk of NAFLD, NAFLD + T2DM and T2DM.

Our findings of lower CTRP5 levels in patients than the controls are in contrast to studies showing that circulating level of CTRP5 was elevated in animal models of diabetes [22]. A similar inconsistency has been also noted for other CTRPs such as CTRP 1 and CTRP3 [12, 15, 18]. The reason behind the discrepancy might be due to differences between animal models and human studies. In details, animal models reflect certain aspects of a disease. For example, in some animal models; insulin resistance predominates, whilst in others β-cell failure is predominant [35]. However, it is well-established that T2DM and NAFLD are heterogeneous conditions that are not attributable to a certain pathophysiological mechanism. In fact, a constellation of interrelated abnormalities is involved in etiology of NAFLD and T2DM [35–37].

Our findings with regard to lack of difference in CTRP5 levels among the patient groups, are in accordance with a recent study by Flehmig et al. in which CTRP5 levels showed no significant difference between obese subjects without diabetes compared to obese patients with diabetes [31]. Although it is difficult to dissect this finding, the possible explanation for our results regarding lack of difference in CTRP5 levels among the patient groups, may lie in fact that there are shared mechanisms in the pathogenesis of NAFLD and T2DM. Specifically, their pathogenesis is the result of common combination of interrelated factors such as inflammation, oxidative stress, obesity and insulin resistance [35, 36]. So, we have adjusted for BMI, WC, hip and WHR in multinomial logistic regression analysis to find out the possible role of obesity in the relationship of CTRP5 with T2DM and NAFLD. Although decrease in circulating levels of CTRP5 remained as a significant risk factor after adjustment for BMI, WC, hip and WHR, but adjustment for BMI and hip resulted in a substantial attenuation of the association between CTRP5 and NAFLD and T2DM. Therefore, it could be deduced that the lack of difference in CTRP5 levels among the patient groups stemmed from the shared risk factors such as obesity in patient groups. However, further clinical studies with a large sample size are required to observe the possible difference among patient groups.

It has been reported that CTRP5 levels, along with other adipokines, are related to HOMA-IR which are in line with our correlation results [31]. However, in contrast to our results, Choi et al. found no association between circulating CTRP5 levels and insulin resistance index and also other cardiometabolic risk factors [30]. Apart from HOMA-IR, the present study also showed inverse correlation between CTRP5 and some of obesity indices. We believe that different inclusion and exclusion criteria used in various human studies and complicated nature of NAFLD and T2DM might justify the discrepancy among human studies.

Insulin resistance and obesity contribute to fatty liver development by altering production of adipokines and cytokines, change in amount of triglyceride synthesis, increasing lipolysis and subsequent delivery of free fatty acids to liver [35, 36, 38].

Although the exact mechanisms by which decreased CTRP5 levels are associated with the increased risk of NAFLD and T2DM cannot be ascertained according to the present study, several possibilities derived from the experimental studies should be considered. First, there is evidence that recombinant CTRP5 enhances GLUT4 translocation and glucose uptake in myocytes [22]. Secondly, treatment of myocytes and liver cells with human CTRP5 increases fatty acid oxidation and concomitantly decreases fatty acid synthesis via activation of AMPK [22, 23]. Since disturbances in fatty acid oxidation and subsequent excessive lipid storage are closely associated with clinicopathological features in NAFLD [39, 40]; it can be speculated that the low CTRP5 level in patients contributes to impaired lipid homeostasis in NAFLD possibly through dysregulation of fatty acid oxidation. Thirdly, it has been reported that globular domain of CTRP5 ameliorates apoptosis and insulin resistance in palmitate-treated myocytes via inhibiting caspase-3 activity, reactive oxygen species accumulation and insulin receptor substrate-1 (IRS-1) reduction [29]. On the other hand, multiple studies have suggested that accumulation of excess saturated fatty acids in muscle cells causes oxidative stress, mitochondrial dysfunction and apoptosis, which all have been linked to insulin resistance [41, 42]. There is also evidence for the association between impaired skeletal muscle fatty acid metabolism and defects in the trafficking and translocation of GLUT4 in skeletal muscle with insulin resistance and obesity [43, 44]. Based on the observations mentioned above, it is tempting to speculate that decreasing CTRP5 levels might contribute to NAFLD and T2DM through obesity and insulin resistance-dependent pathways; however we should not rule out the involvement of other unknown mechanisms.

Another important finding of this study is that circulating CTRP5 level is inversely correlated with LS (a marker of liver fibrosis) [45] and ALT (a marker of hepatic inflammation) [46]. Accordingly, it raises the possibility that decreased CTRP5 levels could be associated with inflammation and hepatocellular damage in NAFLD. However, the measurement of other adipokines and inflammatory markers is warranted to corroborate this concept.

By ROC analyses, CTRP5 demonstrated its value in T2DM diagnosis with good prediction ability. Conversely, CTRP5 alone was not a specific marker enabling us to distinguish NAFLD from T2DM and NAFLD + T2DM; however, astonishing diagnostic capability was obtained after considering LS and FBG. It should be mentioned that future diagnostic studies are required to evaluate diagnostic power of this criteria. Moreover, monitoring of circulating levels of CTRP5 in response to current treatments, along with prospective studies might be helpful in this regard.

Although the current study along with the available literature partly provide novel insights into the role of CTRP5 as a possible contributory factor in the pathogenesis of NAFLD and T2DM, our study was limited by a relatively small sample size. Moreover, our study design is cross-sectional, which precludes us from drawing inferences about causality. Therefore, we acknowledge that further large-scale clinical investigations with longitudinal data are needed to verify our findings. Furthermore, the measurement of the typical adipokine, adiponectin, can provide further support for our results. It should be noted that except for a limited number of studies in humans, the available literature regarding the role of CTRP5 in regulation of lipid and glucose metabolism are mainly restricted to animal studies and in vitro experiments. Therefore, it makes difficult the interpretation of our results and their comparison with other studies.

Collectively, an association between lower circulating levels of CTRP5 and increased risk of NAFLD and T2DM was found in our study. The current study suggests that the association between insulin resistance and NAFLD might mediate at least in part through the effects of low CTRP5. Also, it appears that assessment of CTRP5 together with LS and FBG might be useful to distinguish patients with NAFLD from T2DM and NAFLD + T2DM. However, more experimental studies are necessary to understand the molecular details of the CTRP5 function in regulation of metabolic pathways.

## Additional file

10.1186/s13098-015-0099-z The results of post hoc analysis for anthropometric and laboratory characteristics which were significantly different among the four groups.

## Abbreviations

NAFLD: nonalcoholic fatty liver disease
T2DM: type 2 diabetes mellitus
CTRP5: C1q/TNF-related protein 5
WC: waist circumference
WHR: waist-to-hip ratio
BMI: body mass index
FBG: fasting blood glucose
HOMA-IR: homeostasis model assessment of insulin resistance
TG: triglycerides
TC: total cholesterol
HDL-C: high-density lipoprotein cholesterol
LDL-C: low-density lipoprotein cholesterol
ALT: alanine amino transferase
AST: aspartate amino transferase
ɤ-GT: gamma glutamyl transferase
ALP: alkaline phosphatase
LS: liver stiffness
SBP: systolic blood pressure
DBP: diastolic blood pressure
RBC: red blood cell
WBC: white blood cell
MCV: mean corpuscular volume
RDW: red cell distribution width
APRI: aspartate amino transferase to platelet ratio index
AMPK: AMP-activated protein kinase
SEM: standard error of mean
IQR: inter-quartile range
ROC: receiver operating characteristic
AUC: area under the curve
