# Supplementary material for: The association of circulating levels of complement-C1q TNF-related protein 5 (CTRP5) with nonalcoholic fatty liver disease and type 2 diabetes: a case–control study
Source: Diabetol Metab Syndr. 2015 Nov 25;7:108. doi: 10.1186/s13098-015-0099-z (PMC4660841; doi:10.1186/s13098-015-0099-z)
Supplement: Supplementary file 1 — 10.1186/s13098-015-0099-z The results of post hoc analysis for anthropometric and laboratory characteristics which were significantly different among the four groups. [file 13098_2015_99_MOESM1_ESM.doc]

**Additional file 1: Table S1.** The results of post-hoc analysis for anthropometric and laboratory characteristics which were significantly different among the four groups.

| **p-value** | | | | | | |  | |
| --- | --- | --- | --- | --- | --- | --- | --- | --- |
| **Among**  **the four groups** | **T2DM**  **vs.**  **NAFLD+T2DM** | **NAFLD**  **vs.**  **NAFLD+T2DM** | **NAFLD**  **vs.**  **T2DM** | **Healthy Subjects**  **vs.**  **NAFLD+T2DM** | **Healthy Subjects**  **vs.**  **T2DM** | **Healthy Subjects vs.**  **NAFLD** |  | **Characteristics** |
| <0.001 | 0.01 | 0.49 | 0.7 | <0.001 | 0.1 | 0.001 | cm | WC^#^ |
| <0.001 | 0.002 | >0.999 | 0.1 | <0.001 | >0.999 | 0.01 | cm | Hip^#^ |
| <0.001 | >0.999 | 0.9 | >0.999 | <0.001 | 0.025 | 0.014 | - | WHR^#^ |
| <0.001 | 0.01 | 0.73 | 0.4 | <0.001 | 0. 17 | 0.001 | Kg/m2 | BMI^#^ |
| <0.001 | 0.013 | 0.12 | >0.999 | <0.001 | 0.001 | <0.001 | kPa | LS^#^ |
| <0.001 | 0.92 | <0.001 | <0.001 | <0.001 | <0.001 | 0.103 | mg/dL | FBG* |
| <0.001 | 0.236 | 0.65 | <0.001 | <0.001 | 0.34 | <0.001 | µU/mL | Insulin* |
| <0.001 | 0.43 | 0.68 | 0.998 | <0.001 | 0.012 | <0.001 | - | HOMA-IR* |
| <0.001 | <0.001 | 0.952 | 0.018 | <0.001 | 0.999 | 0.018 | U/L | AST* |
| <0.001 | <0.001 | 0.25 | <0.001 | <0.001 | 0.964 | <0.001 | U/L | ALT* |
| <0.001 | 0.052 | 0.292 | 0.873 | <0.001 | 0.31 | 0.006 | U/L | ɤ-GT* |
| <0. 05 | 0.064 | 0.999 | 0.07 | 0.999 | 0.922 | 0.265 | - | APRI* |

NAFLD, nonalcoholic fatty liver disease ; T2DM, type 2 diabetes mellitus ; WC, waist circumference; WHR, waist-to-hip ratio; BMI, body mass index ;FBG, fasting blood glucose; HOMA-IR, Homeostasis model assessment of insulin resistance; ALT ,alanine amino transferase; AST, aspartate amino transferase ;ɤ-GT, gamma glutamyl transferase ;LS,liver stiffness; APRI, aspartate amino transferase to platelet ratio index.

# Groups were compared using the one-way ANOVA with Bonferroni post hoc test.

* Groups were compared using the Kruskal-Wallis test followed by Mann–Whitney U-test with
Bonferroni correction.
